# Author Correction: A next-generation tumor-targeting IL-2 preferentially promotes tumor-infiltrating CD8^+^ T-cell response and effective tumor control

**DOI:** 10.1038/s41467-020-15532-1

**Published:** 2020-04-01

**Authors:** Zhichen Sun, Zhenhua Ren, Kaiting Yang, Zhida Liu, Shuaishuai Cao, Sisi Deng, Lily Xu, Yong Liang, Jingya Guo, Yingjie Bian, Hairong Xu, Jiyun Shi, Fan Wang, Yang-Xin Fu, Hua Peng

**Affiliations:** 1 Key Laboratory of Infection and Immunity of CAS, Institute of Biophysics, Chinese Academy of Sciences, Beijing, 100101 China; 2 University of Chinese Academy of Sciences, Beijing, 100049 China; 3 Department of Pathology, University of Texas Southwestern Medical Center, Dallas, TX 75235−9072 USA; 4 Department of Biological Sciences, University of Wellesley College, Wellesley, 02481 MA USA; 5 Key Laboratory of Protein and Peptide Pharmaceuticals, CAS Center for Excellence in Biomacromolecules, Institute of Biophysics, Chinese Academy of Sciences, Beijing, 100101 China; 6 Medical Isotopes Research Center and Department of Radiation Medicine, School of Basic Medical Sciences, Peking University, Beijing, China

**Keywords:** Cancer, Immunology

Correction to: *Nature Communications* 10.1038/s41467-019-11782-w, published online 28 August 2019.

The original version of this Article contained an error in the Results section, which incorrectly read ‘Erb-sumIL2 did not make any change for tumor control when used after afatinib treatment. However, the administration of afatinib and the Erb-sumIL2 at the same time effectively control tumor growth and prevent tumor development after being rechallenged with a high dose of tumor cells (Fig. 5f).’ The correct version replaces ‘Erb-sumIL2’ with ‘Her-sumIL2’ in two instances. This has been corrected in both the PDF and HTML versions of the Article.

