# Supplementary material for: A next-generation tumor-targeting IL-2 preferentially promotes tumor-infiltrating CD8+ T-cell response and effective tumor control
Source: Nat Commun. 2019 Aug 28;10:3874. doi: 10.1038/s41467-019-11782-w (PMC6713724; doi:10.1038/s41467-019-11782-w)
Supplement: Supplementary file 1 — Supplementary Information [file 41467_2019_11782_MOESM1_ESM.pdf]

Supplementary Figure 1

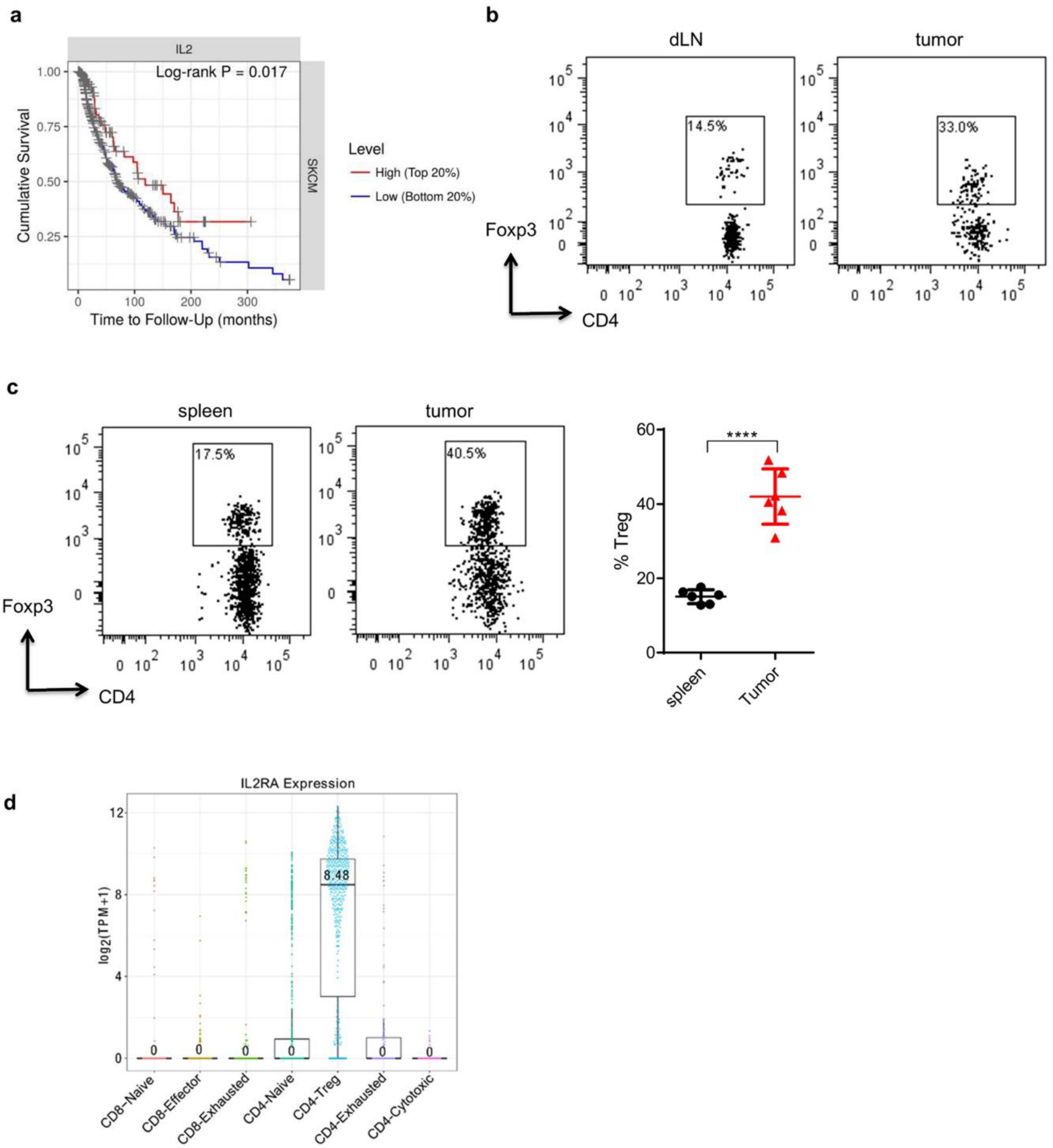

Supplementary Fig. 1 High Treg infiltration may promote tumor progression and limit the therapeutic effect of IL-2. (**a**) The correlation of IL-2 expression level and the survival of skin cutaneous melanoma patients were analyzed by TIMER. (**b**) WT C57BL/6 mice (n=3-5/group) were injected subcutaneously with 5×105 B16F10 cells. The tumor tissues were collected on day 14, and the percentages of Treg cells among CD4+ T cells within tumor tissues and draining lymph nodes were detected by flow cytometry. (**c**) WT C57BL/6 mice (n=5-6/group) were injected subcutaneously with 5×105 MC38 cells. The tumor tissues were collected on day 14, and the percentages of Treg cells among CD4+ T cells within the tumor and spleen tissue was detected by flow cytometry. (**d**) The expression of IL-2 alpha receptor on tumor-infiltrating lymphocytes was analyzed with the published single-cell RNA-sequencing data from hepatocellular carcinoma patients. Unpaired T-tests were used to analyze the other data. ns (not significant), *P<0.05, **P<0.01, ***P<0.0001 and ****P<0.0001. One of two or three representative experiments is shown.

Supplementary Figure 2

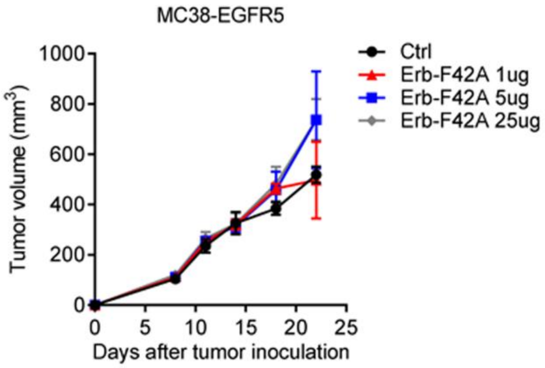

Supplementary Fig. 2 F42A mutant IL-2 is not able to effectively control tumor growth. WT C57BL/6 mice (n=5/group) were injected subcutaneously with $5{\times}10^5$ MC38-EGFR5 and i.p. treated with 1μg, 5μg or 25μg of Erb-F42A IL2 on days 8, 11 and 14. The tumor volume was measured twice a week.

Supplementary Figure 3

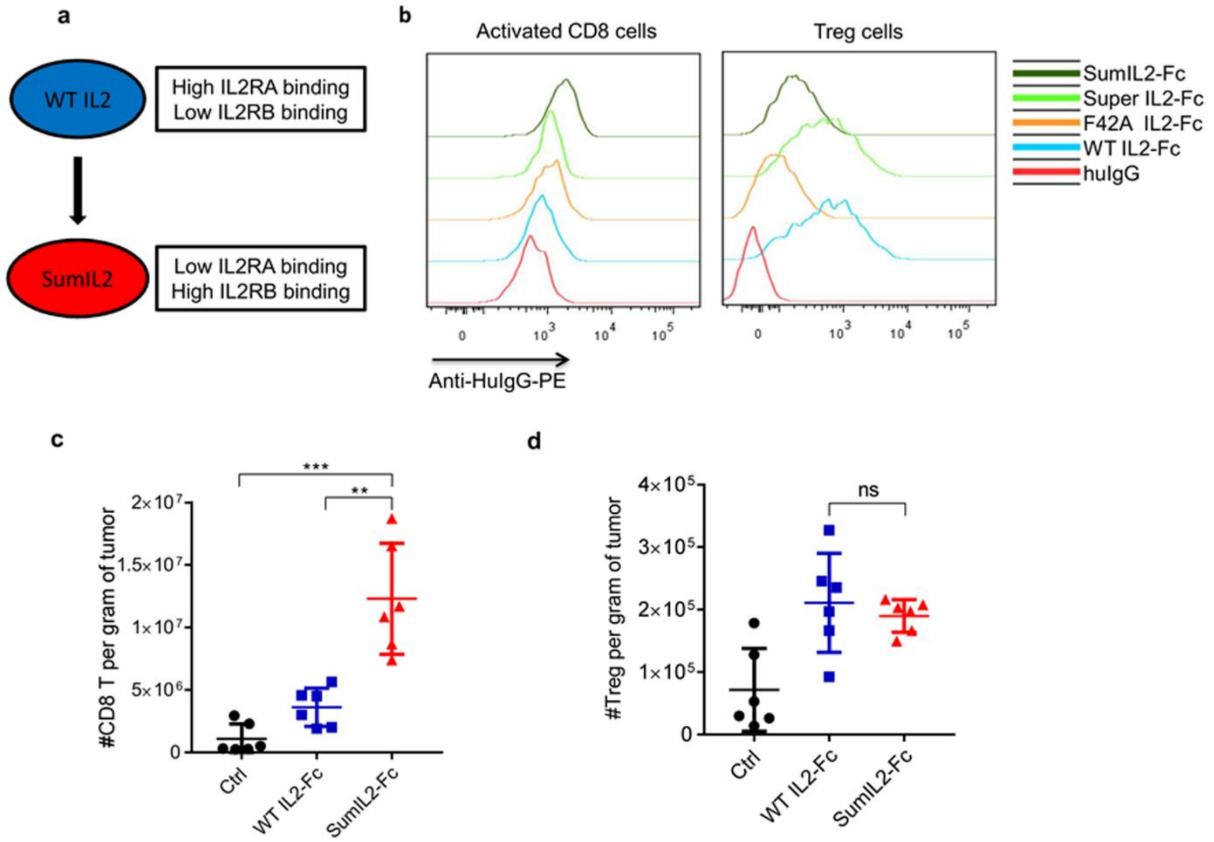

Supplementary Fig. 3 SumIL-2 induces more CD8$^+$ T cells than WT IL-2. (**a**) Super mutant IL2 has F42A, L80F, R81D, L85V, I86V and I92F mutation. **b** Foxp3-GFP mice spleen cells were incubated with human IgG, WT IL2-Fc, F42A IL2-Fc, super IL2-Fc or sumIL2-Fc (0.4μg/ml), followed by anti-huIgG-PE staining. (**c, d**) WT C57BL/6 mice (n=5/group) were injected subcutaneously with 5×10^5 B16F10 and i.t. treated with 5μg of WT IL-2 or sumIL2-Fc on day 9. The tumor tissues were collected on day 12, CD8/CD4 ratio in tumor tissue was analyzed by flow cytometry. The absolute number of CD8$^+$ T cells (c) and Treg cells (d) in tumor tissues were counted.

Supplementary Figure 4

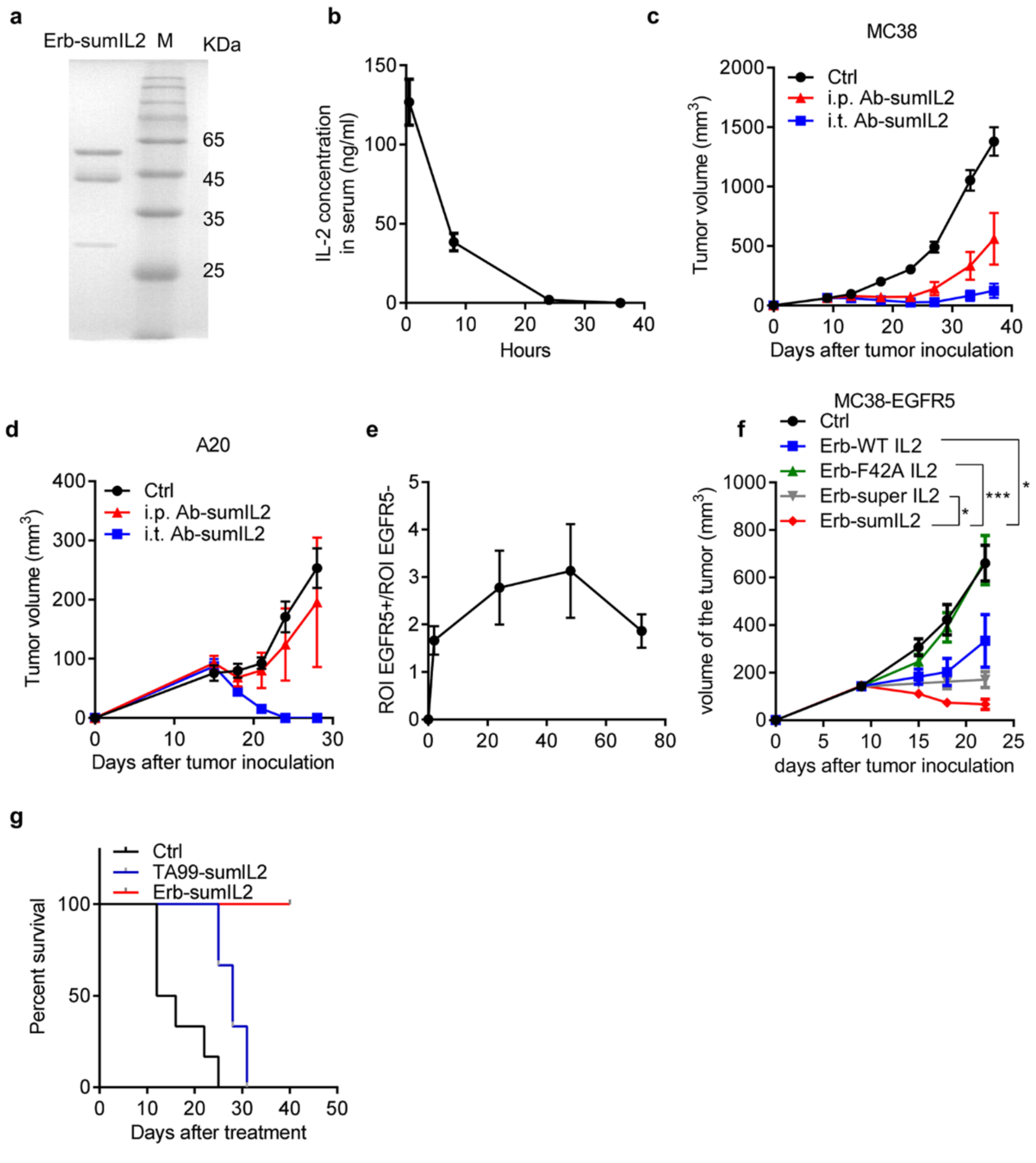

Supplementary Fig. 4. Targeting effect is critical for sumIL-2 therapy. (**a**) The purity of Erb-sumIL2 was assessed by SDS-PAGE. (**b**) The half-life of Erb-sumIL2 in serum was assessed by ELISA. (**c**) C57BL/6 mice (n=5/group) were injected subcutaneously with 5×10^5 of MC38 cells, and 10μg of Erb-sumIL2 was administered (i.p. or i.t.) on days 9, 13, and 17. The growth of the tumor was measured and compared twice a week. (**d**) BALB/c mice (n=3-5/group) were injected subcutaneously with 3×10^6 of A20 cells, 10μg of Erb-sumIL2 was administered (i.p. or i.t.) on days 15 and 18. The growth of the tumor was measured and compared twice a week. (**e**) MC38 (left flank) and MC38-EGFR5 (right flank) tumor-bearing mice were treated with single dose of PBS or 25μg Erb-sumIL2 (i.v.); the ratio fluorescence intensity of MC38-EGFR5 tumor /MC38 tumor post Erb-sumIL2 treatment. (**f**) C57BL/6 mice (n=5/group) were injected subcutaneously with 5×10^5 MC38-EGFR5 cells, and then i.p. treated with 25μg of Erb-IL2 variants on day 9 and the tumor volume was measured. (**g**) C57BL/6 mice were (n=5/group) injected subcutaneously with 5×10^5 of MC38-EGFR5 cells, and then i.v. treated on days 7 and 10 with PBS, 25μg of Erb-sumIL2 or 25μg TA99-sumIL2.

Supplementary Figure 5

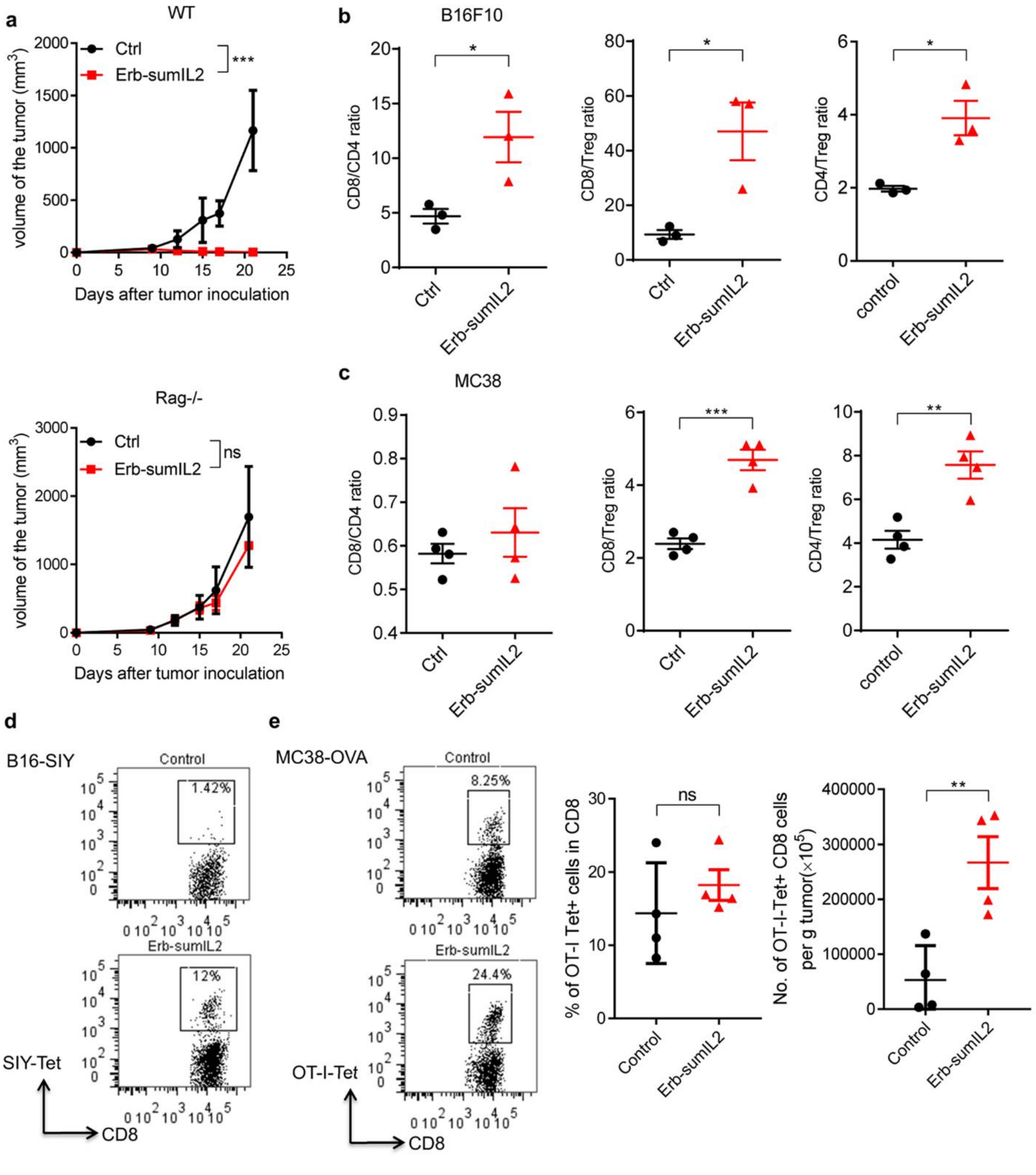

Supplementary Fig. 5. Ab-sumIL2 therapy increases CD8/Treg ratio and promotes CTL response. (**a**) upper panel, C57BL/6 mice were injected subcutaneously with 5×10^5 of B16F10 cells, and then i.t. treated on days 9, 12 and 15 with PBS or 10μg of Erb-muIL2; lower panel, B16F10-bearing Rag1 knockout (KO) mice were i.t. treated with 10μg of Erb-sumIL2 or PBS on the same time point. (**b**) WT C57BL/6 mice were injected subcutaneously with 5×10^5 B16F10 cells and i.t. treated with 10μg Erb-sumIL2 on day 9. The tumor tissues were collected on day 12, and the percentages of $CD45^+$ among total live cells, the ratio of CD8/CD4, CD8/Treg, and CD4/Treg were analyzed by flow cytometry. (**c**) WT C57BL/6 mice were injected subcutaneously with 5×10^5 MC38 cells and i.t. treated with 10μg of Erb-sumIL2 on day 9. The tumor tissues were collected on day 12, and the percentages of $CD45^+$ among total live cells, the ratio of CD8/CD4, CD8/Treg, and CD4/Treg were analyzed by flow cytometry. (**d**) WT C57BL/6 mice were injected subcutaneously with 7.5×10^5 B16-SIY cells and i.t. treated with 10μg of Erb-sumIL2 on day 9. The tumor tissues were collected on day 15. Antigen-specific $CD8^+$ T cells within the tumor were detected using SIY-specific tetramer. The frequency of SIY specific $CD8^+$ T cells among total $CD8^+$ T cells and the number of SIY-specific $CD8^+$ T cells were analyzed by flow cytometry. (**e**) WT C57BL/6 mice were injected subcutaneously with 5×10^5 MC38-OVA cells and i.t. treated with 10μg of Erb-sumIL2 on day 9. The tumor tissues were collected on day 14. Antigen-specific $CD8^+$ T cells in the tumor site were detected using OT-I tetramer. The frequency of OT-I specific $CD8^+$ T cells among total $CD8^+$ T cells and the number of OT-I-specific $CD8^+$ T cells were analyzed by flow cytometry. Two way ANOVA tests were used to analyze the tumor growth data. Unpaired T-tests were used to analyze the other data. ns (not significant), *P<0.05, **P<0.01, ***P<0.0001 and ****P<0.0001. One of two or three representative experiments is shown.

Supplementary Figure 6

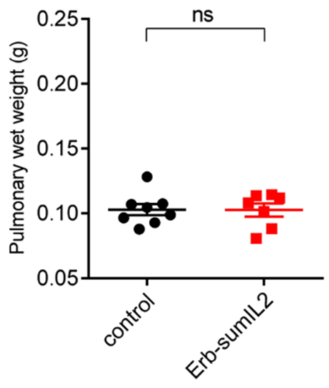

Supplementary Fig. 6 SumIL-2 therapy does not cause pulmonary edema. MC38-EGFR5 bearing mice were i.p. treated with 25μg Erb-sumIL2 for three times; the control group was injected with PBS. The wet weight of lung tissue was measured on day 7 post-treatment. Pulmonary wet weight was measured to assess adverse toxic effects following Erb-sumIL2 treatment and was determined by weighing lungs before and after drying. Unpaired T-tests were used to analyze the data.

Supplementary Table 1

Antibodies used in this study.

Antibodies	Source	Identifier
InVivoMab anti-mouse CD4 (GK1.5)	Produced in the lab	
InVivoMab anti-mouse CD8 (2.43)	Produced in the lab	
InVivoMab anti-mouse NK1.1(PK136)	Produced in the lab	
InVivoMab anti-mouse PD-L1 (10F.9G2)	BioXcell	Cat# BE0101
InVivoMab anti-mouse Ly6G (1A8)	BioXcell	Cat# BP0075
Anti-mouse CD45 (FACs, 30-F11)	Invitrogen	Cat# 56-0451-82
Anti-mouse CD4 (FACs, RM4-5)	Invitrogen	Cat# 45-0042-82
Anti-mouse CD8 (FACs, 53-6.7)	Invitrogen	Cat# 47-0081-82
Anti-Foxp3 (FACs, FJK-16s)	Invitrogen	Cat# 12-5773
Anti-mouse CD3Ɛ (FACs, 145-2C11)	Invitrogen	Cat# 25-0031-82
Anti-mouse CD11b (FACs, M1/70)	Invitrogen	Cat# 45-0112
Anti-mouse CD274 (FACs, MIH5)	Invitrogen	Cat# 12-5982-82
Anti-mouse CD279 (FACs, J43)	Invitrogen	Cat# 17-9985-82
Anti-mouse CD185 (FACs, SPRCL5)	Invitrogen	Cat# 13-7185-82
Anti-mouse CD11c (FACs, N418)	Biolegend	Cat# 117310
Anti-mouse F4/80 (FACs, 8M8)	Biolegend	Cat# 123113
Anti-mouse I-A/I-E (FACs, M5/114.15.2)	Biolegend	Cat# 107628
iTAg Tetramer/PE-H-2 Kb OVA (SIINFEKL)	MBL	Cat# TB-5001-1
iTAg Tetramer/PE-H-2 Kb SIY (SIYRYYGL)	MBL	Cat# TS-M008-P
