# Supplementary material for: A next-generation tumor-targeting IL-2 preferentially promotes tumor-infiltrating CD8+ T-cell response and effective tumor control
Source: Nat Commun. 2019 Aug 28;10:3874. doi: 10.1038/s41467-019-11782-w (PMC6713724; doi:10.1038/s41467-019-11782-w)
Supplement: Supplementary file 2 — Reporting Summary [file 41467_2019_11782_MOESM2_ESM.pdf]

nature research

Corresponding author(s): Dr. Yang-Xin Fu

Last updated by author(s): Jul 23, 2019

Reporting Summary

Nature Research wishes to improve the reproducibility of the work that we publish. This form provides structure for consistency and transparency in reporting. For further information on Nature Research policies, see Authors & Referees and the Editorial Policy Checklist.

Statistics

For all statistical analyses, confirm that the following items are present in the figure legend, table legend, main text, or Methods section.

n/a	Confirmed	
☐	☒	The exact sample size (n) for each experimental group/condition, given as a discrete number and unit of measurement
☐	☒	A statement on whether measurements were taken from distinct samples or whether the same sample was measured repeatedly
☐	☒	The statistical test(s) used AND whether they are one- or two-sided<br>*Only common tests should be described solely by name; describe more complex techniques in the Methods section.*
☐	☒	A description of all covariates tested
☐	☒	A description of any assumptions or corrections, such as tests of normality and adjustment for multiple comparisons
☐	☒	A full description of the statistical parameters including central tendency (e.g. means) or other basic estimates (e.g. regression coefficient) AND variation (e.g. standard deviation) or associated estimates of uncertainty (e.g. confidence intervals)
☒	☐	For null hypothesis testing, the test statistic (e.g. F, t, r) with confidence intervals, effect sizes, degrees of freedom and P value noted<br>*Give P values as exact values whenever suitable.*
☒	☐	For Bayesian analysis, information on the choice of priors and Markov chain Monte Carlo settings
☒	☐	For hierarchical and complex designs, identification of the appropriate level for tests and full reporting of outcomes
☒	☐	Estimates of effect sizes (e.g. Cohen's d, Pearson's r), indicating how they were calculated

Our web collection on statistics for biologists contains articles on many of the points above.

Software and code

Policy information about availability of computer code

Data collection	BD FACSDiva software, NanoDrop, IVIS Spectrum, Tanon MP
Data analysis	GraphPad Prism software 7.0, FlowJo software v7.6, LivingImage

For manuscripts utilizing custom algorithms or software that are central to the research but not yet described in published literature, software must be made available to editors/reviewers. We strongly encourage code deposition in a community repository (e.g. GitHub). See the Nature Research guidelines for submitting code & software for further information.

Data

Policy information about availability of data

All manuscripts must include a data availability statement. This statement should provide the following information, where applicable:

- Accession codes, unique identifiers, or web links for publicly available datasets
- A list of figures that have associated raw data
- A description of any restrictions on data availability

The data that support the findings of this study are available from the corresponding author on reasonable request.

Field-specific reporting

Please select the one below that is the best fit for your research. If you are not sure, read the appropriate sections before making your selection.

☒ Life sciences ☐ Behavioural & social sciences ☐ Ecological, evolutionary & environmental sciences

For a reference copy of the document with all sections, see nature.com/documents/nr-reporting-summary-flat.pdf

Life sciences study design

All studies must disclose on these points even when the disclosure is negative.

Sample size	Sample size were determined from the similar experiments in the former publications of the group.
Data exclusions	No data were excluded.
Replication	The performed replications were successful.
Randomization	Mice in this study were matched with age and tumor size, and were randomly allocated to the different groups.
Blinding	Collection of animal samples was not blinded. However, data analyzed were blinded.

Reporting for specific materials, systems and methods

We require information from authors about some types of materials, experimental systems and methods used in many studies. Here, indicate whether each material, system or method listed is relevant to your study. If you are not sure if a list item applies to your research, read the appropriate section before selecting a response.

Materials & experimental systems

n/a	Involved in the study	
☐	☒	Antibodies
☐	☒	Eukaryotic cell lines
☒	☐	Palaeontology
☐	☒	Animals and other organisms
☒	☐	Human research participants
☒	☐	Clinical data

Methods

n/a	Involved in the study	
☒	☐	ChIP-seq
☐	☒	Flow cytometry
☒	☐	MRI-based neuroimaging

Antibodies

Antibodies used	InVivoMab anti-mouse CD4 (GK1.5); InVivoMab anti-mouse CD8 (2.43); InVivoMab anti-mouse NK1.1(PK136); all Mabs above were produced in the Lab. InVivoMab anti-mouse PD-L1 (10F.9G2), BioXcell, BE0101; InVivoMab anti-mouse Ly6G (1A8), BioXcell, BP0075; Anti-CD45 (FACs, 30-F11), Invitrogen, 56-0451-82; Anti-CD4 (FACs, RM4-5), Invitrogen, 45-0042-82; Anti-CD8 (FACs, 53-6.7), Invitrogen, 47-0081-82; Anti-Foxp3 (FACs, FJK-16s), Invitrogen, 12-5773; Anti-mouse CD3Ɛ (FACs, 145-2C11), Invitrogen 25-0031-82; Anti-mouse CD11b (M1/70), Invitrogen, 45-0112; Anti-mouse CD11c (N418), Biolegend, 117310; Anti-mouse F4/80 (8M8), Bioleend, 123113; Anti-mouse I-A/I-E (M5/114.15.2), Biolegend, 107628; Anti-mouse CD274 (MIH5), Invitrogen, 12-5982-82; Anti-mouse CD279 (J43), Invitrogen, 17-9985-82; Anti-mouse CD185 (SPRCL5) Invitrogen, 13-7185-82; iTAg Tetramer/PE-H-2 Kb OVA (SIINFEKL), MBL, TB-5001-1; iTAg Tetramer/PE-H-2 Kb SIY (SIYRYYGL), MBL, TS-M008-P
Validation	Antibody validations were performed by suppliers and have been published by our group and others

Eukaryotic cell lines

Policy information about cell lines

Cell line source(s)	ATCC
Authentication	The cell lines obtained from ATCC with responsive authentication and characterization.
Mycoplasma contamination	All the cell lines used in this study was tested negative for mycoplasma contamination.
Commonly misidentified lines (See ICLAC register)	None

Animals and other organisms

Policy information about studies involving animals; ARRIVE guidelines recommended for reporting animal research

Laboratory animals	Wild-type (WT) C57BL/6NCrl and BALB/c mice were purchased from the Vital River Laboratories, and C57BL/6 Rag1 KO mice were purchased from model animal research center of Nanjing university. Foxp3-GFP mice (BALB/c background) were kindly provided by Dr. Yong Zhao at the Institute of Zoology, CAS. All mice were maintained under specific pathogen-free conditions in the animal facility of the Institute of Biophysics.

Wild animals	The study did not involve wild animals.
Field-collected samples	The study did not involve field-collected samples
Ethics oversight	This study has been approved by the Institutional Animal Care and Use Committee of the Institute of Biophysics.

Note that full information on the approval of the study protocol must also be provided in the manuscript.

Flow Cytometry

Plots

Confirm that:

☒ The axis labels state the marker and fluorochrome used (e.g. CD4-FITC).

☒ The axis scales are clearly visible. Include numbers along axes only for bottom left plot of group (a 'group' is an analysis of identical markers).

☒ All plots are contour plots with outliers or pseudocolor plots.

☒ A numerical value for number of cells or percentage (with statistics) is provided.

Methodology

Sample preparation	Tumor tissues were Cut into small pieces with scissors (about 1mm*1mm*1mm) and digested in the digested buffer (1mg/ml collagenase IV and 0.05mg/ml DNase I in RPMI medium) at 37 degree for 40 minutes with the speed at 145RPM. Add 10ml 10% FBS RPMI medium to stop the digestion, and spin down and resuspend the pellet. Tumor cell suspension was blocked with the anti-CD16/32 antibody (clone 2.4G2) for 30 min, and then incubated with indicated antibody for 30 min at 4 °C in the dark. DAPI or LIVE/DEAD™ fixable yellow dye (ThermoFisher) was used to exclude dead cells.
Instrument	FACSFortessa flow cytometer (BD)
Software	BD FACSDiva software, FlowJo software v7.6
Cell population abundance	Physical parameter and LIVE/DEAD™ fixable yellow dye (ThermoFisher) was used to exclude dead cells. Positive populations were defined using not stained cells as reference. Isotype controls were used to confirm the specificity of the staining. In some experiments, the percentage of the relevant cell populations is shown in the supplementary figure.
Gating strategy	DAPI or LIVE/DEAD™ fixable yellow dye (ThermoFisher) was used to exclude dead cells. Positive populations were defined using not stained cells as reference. Isotype controls were used to confirm the specificity of the staining.

☒ Tick this box to confirm that a figure exemplifying the gating strategy is provided in the Supplementary Information.
